# Update on human genetic susceptibility to COVID-19: susceptibility to virus and response

**DOI:** 10.1186/s40246-021-00356-x

**Published:** 2021-08-25

**Authors:** Vito Luigi Colona, Vasilis Vasiliou, Jessica Watt, Giuseppe Novelli, Juergen K. V. Reichardt

**Affiliations:** 1 Department of Biomedicine and Prevention, “Tor Vergata” University of Rome, 00133 Rome, Italy; 2 Department of Environmental Health Sciences, School of Public Health, Yale University, New Haven, USA; 3 College of Public Health, Medical and Veterinary Sciences, James Cook University, Smithfield, QLD Australia; 4 IRCCS Neuromed, Pozzilli, IS Italy; 5 Department of Pharmacology, School of Medicine, University of Nevada, Reno, NV 89557 USA; 6 Australian Institute of Tropical Health and Medicine, James Cook University, Smithfield, QLD 4878 Australia

## Introduction

Over the past year and a half, SARS-CoV-2, the etiological agent of the COVID-19 pandemic, led to a total of almost 200 million cases, causing more than 4 million of deaths globally (Johns Hopkins University, CSSE) [1].

While we are facing rising daily hospitalizations (https://ourworldindata.org/covid-hospitalizations, accessed on July 31, 2021) [2], attributable to novel emerging variants of the virus [3, 4], we also observe a decrease in both hospitalizations due to severe forms of the disease and deaths in several parts of the world, thanks to the launch of massive vaccination campaigns [2]. To date, 4 billion vaccine doses have been administered [1]. Despite of the efforts of global organizations to face this health emergency, including the COVAX plan which aims to achieve the vaccination coverage in developing countries [5], we are still far from reaching the desired results and the end of this pandemic especially in emerging countries.

As we discussed in our recent review on “COVID-19 one year into the pandemic: from genetics and genomics to therapy, vaccination, and policy” [6], vaccines represent one of the most valuable aid to halt the SARS-CoV-2 spread. The emergence of novel variants of concern (VOC) aroused concern among the scientific community, since they are associated with a rise of viral transmissibility [7], and with a reduction in the therapeutic response to both monoclonal antibodies and antibody activity in vaccinated individuals [8]. Nevertheless, results arising from the analysis of vaccine coverage against the emerging Delta variant are promising [9].

It is known that the mRNA vaccines, both BNT162b2 (Pfizer/BioNTech) and mRNA-1273 (Moderna), can potentially be implemented to match the need of a response against SARS-CoV-2 mutations. For this reason, it is crucial to increase the genomic surveillance in the different departments of public health systems all over the world [10].

In the same publication [6], we arrive at the conclusion that not only the virus, but significantly also the synergic relationship with the host represents the core of the understanding of mechanisms underpinning the infectious cycle, transmission, resistance and susceptibility to SARS-CoV-2. In addition, we also expressed concern about effects that environmental pollution may exert on susceptibility to SARS-CoV-2 by diminishing immune responses. We are aware that increased knowledge of this aspects is fundamental to unveil the clinical course and a more targeted therapeutical approach for patients affected by COVID-19.

In this editorial, we focus on genetic and genomics susceptibility factors to COVID-19, and we aim to summarize the current knowledge in the literature providing an updated, easy to consult and constantly revised tool, through an update of Table 2 from our recent review [6].

## Discussion

Going beyond the one-year landmark into the pandemic caused by SARS-CoV-2, we perceive that an in-depth analysis of human genetic susceptibility to the severity of the disease is becoming increasingly crucial.

Table 1 summarizes ultra-rare, rare and common human variants, haplotypes, and susceptibility gene polymorphisms detected in several studies, through various approaches [11–17].

**Table 1:** Genetic risk factors for severe COVID-19

SARS-CoV-2 susceptibility gene variant or haplotype	Risk estimated [OR]	Frequency [MAF]	References
*TLR3*, *UNC93B1*, *TICAM1*, *TBK1*, *IRF3*, *IRF7*, *IFNAR1*, *IFNAR2* (autosomal-dominant model)	9	< 0.001	Zhang et al. [20]
*IRF7*, *IFNAR1* (autosomal-recessive model)	> 50	< 0.001	Zhang et al. [20]
rs769208985—missense variant of *FURIN*	N.A	< 0.001	Latini et al. [71]
rs150892504—missense variant of *ERAP2*	N.A	0.002	Hu et al. [60]
rs138763430—missense variant of *BRF2*	N.A	0.002	Hu et al. [60]
rs147149459—missense variant of *ALOXE3*	N.A	0.002	Hu et al. [60]
rs117665206—missense variant of *TMEM181*	N.A	0.006	Hu et al. [60]
rs114363287—missense variant of *TMPRSS2*	N.A	0.006	Latini et al. [71]
rs61756766—missense variant of *TNFRSF13C*	12.3	0.008	Russo et al. [61]
rs7626962—missense variant of *SCN5A*	8.7	0.008	SeyedAlinaghi et al. [62]
rs1805128—missense variant of *KCNE1*	9.0	0.009	SeyedAlinaghi et al. [62]
HLA-DRB*27:07	N.A	0.02	Novelli et al. [51]
rs72711165—intronic variant of *TMEM65*	1.2	0.02	COVID-19 H.G.I. [66]
rs115492982—intronic variant of *MRPS21*	2.5	0.02	Dite et al. [63]
rs74956615—3'UTR variant of *TYK2*	1.6	0.03	Pairo-Castineira et al. [11]
rs2034831—intronic variant of *ITGA4*	1.2	0.05	Dite et al. [63]
rs76374459—intronic variant of *LZTFL1*	1.2	0.05	Dite et al. [63]
rs35652899—intronic variant of *LZTFL1*	1.2	0.05	Dite et al. [63]
rs10490770—intronic variant of *LZTFL1*	2.0	0.06	COVID-19 H.G.I. [66]
rs333—CCR5-Δ32	0.7	0.07	Cuesta-Llavona et al. [76]
rs73064425—intronic variant of *LZTFL1*	2.1	0.08	Pairo-Castineira et al. [11], Ellinghaus et al. [23]
rs11385942—intronic variant of *LZTFL1*	1.8	0.07	Ellinghaus et al. [23]
rs1886814—intronic variant of *FOXP4*	1.3	0.07	COVID-19 H.G.I. [66]
rs76488148—intronic variant of *GYG1*	1.3	0.07	Dite et al. [63]
rs2271616—5'UTR variant of *SLC6A20*	1.1	0.08	COVID-19 H.G.I. [66]
HLA-DQB1*06:02	N.A	0.08	Novelli et al. [51]
rs143334143—intronic variant of *CCHCR1*	1.9	0.09	Pairo-Castineira et al. [11]
HLA-DRB1*15:01	N.A	0.10	Novelli et al. [51]
rs12252:G allele of *IFITM3*	2.2	0.13	Alghamdi et al. [52]
rs4801778—intronic variant of *PLEKHA4*	1.0	0.16	COVID-19 H.G.I. [66]
rs6598045—5'UTR variant of *IFITM3*	N.A	0.19	Kim et al. [53]
rs429358—missense variant of *APOE*	2.3–2.4	0.20	Kuo et al. [65]
rs12610495—intronic variant of *DPP9*	N.A	0.25	Moon et al. [41]
rs12329760—intronic variant of *TMPRSS2/MX1*	0.9	0.25	Andolfo et al. [72]
rs2298661—missense variant of *TMPRSS2/MX1*	0.9	0.25	Andolfo et al. [72]
rs3787946—intronic variant of *TMPRSS2/MX1*	0.9	0.28	Andolfo et al. [72]
rs9983330—intronic variant of *TMPRSS2/MX1*	0.9	0.28	Andolfo et al. [72]
rs9380142—3'UTR variant of *HLA-G*	13	0.29	Pairo-Castineira et al. [11]
rs2109069—intronic variant of *DPP9*	1.4	0.33	Pairo-Castineira et al. [11], COVID-19 H.G.I. [66]
rs9985159—intronic variant of *TMPRSS2/MX1*	0.9	0.33	Andolfo et al. [72]
Rs75603675—missense variant of *TMPRSS2*	N.A	0.36	Latini et al. [71]
rs1405655—intronic variant of *NR1H2*	1.1	0.37	COVID-19 H.G.I. [66]
rs12329760—missense variant of *TMPRSS2*	0.9	0.39	Hou et al. [73]
rs657152—intronic variant of *ABO*	1.3	0.41	Ellinghaus et al. [23]
rs677800—intronic variant of *ABO*	N.A	0.55	Moon et al. [41]
rs6020298—intronic variant of *TMEM189-UBE2V1*	1.2	0.58	Wang et al. [74]
rs10735079—intronic variant of *OAS1/3*	1.3	0.64	Pairo-Castineira et al. [11]
rs8065800—intronic variant of *MAPT*	1.7	0.65	COVID-19 H.G.I. [66]
rs10774671—intronic, splicing variant of *OAS1*	1.1	0.67	COVID-19 H.G.I. [66]
rs13050728—intronic variant of *IFNAR2*	0.9	0.69	COVID-19 H.G.I. [66]
rs2236757—intronic variant of *IFNAR2*	1.3	0.71	Pairo-Castineira et al. [11]
rs3131294—intronic variant of *NOTCH4*	1.5	0.90	Pairo-Castineira et al. [11]
HLA-A*11	N.A	N.A	Fricke-Galindo et al. [54]
HLA-A*11:01:01:01	2.3	N.A	Khor et al. [56]
HLA-A*25:01	N.A	N.A	Fricke-Galindo et al. [54]
HLA-B*46:01	2.1	N.A	Lin et al. [53], Fricke-Galindo et al. [54]
HLA-B*51:01	N.A	N.A	Fricke-Galindo et al. [54]
HLA B*54:01	5.4	N.A	Lin et al. [55]
HLA-C*01	N.A	N.A	Fricke-Galindo et al. [54]
HLA-C*01:02	N.A	N.A	Fricke-Galindo et al. [54]
HLA-C*05	N.A	N.A	Fricke-Galindo et al. [54]
HLA-C*12:02:02:01-HLA*52:01:02:02	2.3	N.A	Khor et al. [56]
HLA-C*14:02	N.A	N.A	Fricke-Galindo et al. [54]
HLA-C*17	N.A	N.A	Bonaccorsi et al. [57]
HLA-DQB1*04	N.A	N.A	Fricke-Galindo et al. [54]
HLA-DQB1*08	N.A	N.A	Fricke-Galindo et al. [54]
HLA-E*0101/0103	2.1–2.7	N.A	Vietzen et al. [58]
*KLRC2*^del^	2.6–7.1	N.A	Vietzen et al. [58]
*ACE1 I/D* genotype	2.5	N.A	Verma et al. [69]
*C9orf72* with HREs > 10 units	2.4	N.A	Zanella et al. [64]
c.2129_2132del, p.Gln710Argfs*18—frameshift variant of *TLR7*	N.A	N.A	van der Made et al. [42]
c.2383G > T, p.Val795Phe—missense variant of *TLR7*	N.A	N.A	van der Made et al. [42]
c.644A > G, p.Asn215Ser—missense variant of *TLR7*	N.A	N.A	Solanich et al. [43]
c.2797 T > C, p.Trp933Arg—missense variant of *TLR7*	N.A	N.A	Solanich et al. [43]
c.901 T > C, p.Ser301Pro—missense variant of *TLR7*	N.A	N.A	Fallerini et al. [44]
c.3094G > A, p.Ala1032Thr— missense variant of *TLR7*	N.A	N.A	Fallerini et al. [44]
c.2759G > A, p.Arg920Lys—missense variant of *TLR7*	N.A	N.A	Fallerini et al. [44]
c.863C > T, p.Ala288Val—missense variant of *TLR7*	N.A	N.A	Fallerini et al. [44]
c.1342C > T, p.Ala448Val—missense variant of *TLR7*	N.A	N.A	Fallerini et al. [44]
c.655G > A, p.Val219Ile—missense variant of *TLR7*	N.A	N.A	Fallerini et al. [44]
rs140312271—missense variant of *ACE2*	N.A	N.A	Novelli et al. [75]

*MAF* Major Allele Frequency; *N.A.* Not Applicable; *OR* Odds Ratio

Genome-wide association studies (GWAS) led to the identification of susceptibility alleles in several genes, which are linked to severe and/or life-threatening phenotypes. So far, some of the risk values turned out to be too low (OR < 2; odds ratio) to be considered as predictive genomic markers. Nevertheless, it cannot be excluded that the additive effect of these alleles could contribute to a polygenic risk score analysis [18]. On the other hand, high penetrance alleles of genes encoding for proteins involved in crucial homeostasis pathways might be useful for patient stratification and may potentially impact the prognostic field and the pharmacological treatment [19, 20]. However, we have to consider that in a polygenic and multifactorial disease such as COVID-19, several genetic and epigenetic factors are able to regulate phenotypic expression, complicating a possible genotype–phenotype correlation analysis. Pathway analyses are to be considered. In fact, genes encoding for proteins involved in molecular mechanisms of innate immunity and humoral response were among the first candidate genes to be analyzed [19–21].

Innate immunity represents the first immediate, non-specific and autonomous defense line of our immune system and plays a fundamental role against pathogens, including SARS-CoV-2 [22]. Innate immune system deficits can be the basis of heterogeneity in clinical phenotypes and of the outcome of patients affected by COVID-19. This evidence is supported not only by recent discoveries in the genetic field [11, 20, 23], but also by the description of novel mechanisms for the interaction network between viral proteins and host factors [24]. As mentioned in our recent review [6], identifying the functional role of rare variants will allow us to unveil the pathogenetic mechanisms, but also to improve the efficiency of predictive tests for the benefit of a better therapeutical approach in the context of a personalized medicine. Environmental factors also need to be considered.

Zhang et al. [20] and Bastard et al. [21] described the presence of alleles underpinning altered type 1b Interferon (IFN-1b) response in critical patients, and proved that anti-interferon antibodies in the serum of affected individuals in critical conditions are capable of halting the IFN activity. Furthermore, the presence of these antibodies in patients with severe phenotype that required hospitalization to intensive care units (ICU) was successfully proved, establishing a correlation with an high mortality rate [25]. In a further study [26] a significant difference in the amount of inflammatory cytokines emerged in a comparison between cells collected through bronchoalveolar lavage (BAL) in SARS-CoV-2 positive patients and cells collected from patients afflicted by pneumonia of bacterial or viral origin. This shows us how the virus-triggered pyroptosis induces a profound alteration of the immune response, which is also and above all influenced by the host genetic background. A work [19] coordinated by the international consortium CHGE (Covid Human Genetic Effort, https://www.covidhge.com/about) shows that 3% of COVID-19 patients who harbor Loss-of-Function (LoF) variants in loci of genes involved in the response to viral infection pathway has a severe/life-threatening phenotype, estimating an OR around 9 in an autosomal dominant model, and between 50 and 100 in an autosomal recessive model (Table 1).

In addition, further evidence shed a light on the ability of SARS-CoV-2 to adopt several strategies to antagonize the IFNs system, with a subsequent reduction in the type I IFN response [27–29]. It is known, indeed, that the elaborate IFNs system exerts its function in various aspects of the immune response, both innate and adaptive, also filling a role in the immunosurveillance [30]. It follows that congenital defects of this network, together with several comorbidities, are responsible for an inauspicious clinical course and can be counted among severity and susceptibility alleles with an higher-risk estimation impact (Table 1).

In an interesting association study from the 2020 performed in the United Kingdom, based on a cohort of 2,244 severely affected patients, Pairo-Castineira et al. [11] identified novel potential susceptibility alleles listed below and shown in Table 1: rs74956615 (3’UTR variant of *TYK2*; OR = 1.6; AF = 0.03); rs143334143 (intronic variant of *CCHCR1*; OR = 1.9; AF = 0.09); rs9380142 (3’UTR variant of HLA-G; OR = 13; AF = 0.29); rs2109069 (intronic variant of *DPP9*; OR = 1.4; AF = 0.33); rs10735079 (intronic variant of *OAS1/3*; OR = 1.3; AF = 0.64); rs2236757 (intronic variant of *IFNAR2*; OR = 1.3; AF = 0.71); rs3131294 (intronic variant of *NOTCH4*; OR = 1.5; AF = 0.90); rs73064425 (intronic variant of *LZTFL1*; OR = 2.1; AF = 0.08). In this study, a reduced expression of *IFNAR2* and a high expression of *TYK2*, both involved in the IFN type I immune response pathway, have been associated with severe forms of COVID-19 [11]. Particularly, the putative role of *TYK2*, a tyrosine kinase belonging to the Janus proteins family (JAKs), in the disease severity progression allowed some researchers to speculate on a possible use of specific class of JAK inhibitor compounds. This class enlists the monoclonal antibody baricitinib. Several studies reported the efficiency of the compound whether in association or not with a steroid therapy, and/or with other compounds (*e.g.*, Remdesivir) [31–36].

*DPP9* is located on chromosome 19p13.3 and encodes for a serine protease, the dipeptidyl peptidase 9, and is involved in several stages of the inflammatory response [37–39]. Variants affecting this locus are known to be associated with idiopathic pulmonary fibrosis [40]. From our rapid literature review, a second intronic variant of *DDP9* gene has been accounted among the risk allele for COVID-19 (rs12610495, Table 1) [41]. The latter is another example of how a predisposition to an altered pathophysiology, due to a specific genetic background of the host, might lead the patient through a poor prognosis.

It is noteworthy the discovery of two rare and deleterious germinal variants of the *TLR7* gene in two couples of young male siblings with no reported comorbidities and displaying a severe COVID-19 phenotype: the frameshift variant with maternal segregation c.2129_2132del, p.Gln710Argfs*18 and the missense variant c.2383G > T, p.Val795Phe (Table 1) [42]. The alteration of the response to type I and type II IFN subsequent to imiquimod administration, a TLR7 receptor agonist, confirmed the importance of the maintenance of TLR pathway in COVID-19 pathogenesis [42, 43]. These results found confirmation in a recent independent cohort study performed in Italy, which highlighted the presence of deleterious variants of *TLR7* in 2.1% of severely affected males in comparison with asymptomatic individuals [44]. The specific missense variants are reported in the third section of Table 1. It is known that complete deficit of TLR7 is extremely rare because members of TLRs family (TLR3, TLR7, TLR8 e TLR9) carry out specific and non-redundant activities for the host survival [45, 46]. Variants of the *TLR7* gene are associated with the immune response against single-stranded RNA virus and to the onset of autoimmune disease such as systemic lupus erythematosus (SLE) [47].

Several scientific contributions focused on the potential relevance of HLA complex polymorphisms for SARS-CoV-2 susceptibility (Table 1) [23, 48–58] and for the disease severity extent [59].

The estimate of predictive models deserves a separate discussion. Due to the availability of international biobanks, it has been possible to identify and confirm novel susceptibility loci for COVID-19 [60–66]. Thirteen novel susceptibility loci have been linked to several aspects of SARS-CoV-2 infection as a result of a recent meta-analysis performed by COVID-19 Host Genetics Initiative (COVID-19 H.G.I.) [67]. However, many of those overlap previously reported associations [11, 23, 41], among which the *ABO* loci variants (Table 1) [23, 41] and the 5’UTR variant of *SLC6A20* (rs2271616; OR = 1.1; AF = 0.08. Table 1), which seem to have a major role in infection susceptibility and in the negative progression of the disease [67].

It has been proved that expression levels of genes encoding for proteins involved in the viral uptake (*e.g.,* ACE2, TMPRSS2, FURIN) change with age, which depicts a biological rationale for the broad phenotypic spectrum of COVID-19 [68]. Age and sex, indeed, represent non-genetic factors that primarily influence disease severity [69], but not exclusively. Verma et al. [70] reported a correlation between *ACE I/D* (OR = 2.5. Table 1) polymorphism and a cluster of patients affected by diabetes mellitus and high blood pressure.

Blume et al. [71] identified a new isoform of ACE2, mainly expressed in rhino-oropharynx mucosa. This is downregulated in response to IFN, but not from SARS-CoV-2. For this reason, we think that characterization of *ACE2* promoter functional elements and its regulatory factors represents a fundamental support for the understanding of viral molecular mechanisms. Several studies showed that the presence of rare or novel variants of those genes might be responsible for diversification of the response (Table 1) [72–76].

Last but not least, nowadays, the role of co-receptor 5 (CCR5) deletion 32 in coronavirus susceptibility is still controversial [77–80].

## Conclusions

The current COVID-19 pandemic has had, and will continue to have, a significant impact on humanity. We are aware that not only will society change, but also the way of approaching science. As scientists, we can only be proud of how so many highly skilled research laboratories have been able to put aside their differences to cooperate and achieve extraordinary results [6], opening the way to translational research [81] and personalized medicine [82].

Furthermore, it is clear that precision medicine has the potential to reduce side effects and to reduce hospitalization costs and duration [83]. Studying new therapeutic approaches [84–88], unveiling new molecular mechanisms [88–90], understanding the implications of possible susceptibility alleles in the exposed population [91, 92], will allow us to broaden our contribution to the fight against the current coronavirus outbreak and against upcoming agents which, inevitably, will show up in the near future [93]. With this goal, we make the commitment to constantly update the tool provided within this paper with new data, in order to have an even more precise, in-depth and proactive overview.

## Data Availability

Data sharing is not applicable to this article as no datasets were generated or analyzed during the current study.

## Abbreviations

ACE2: Adenosine-Converting Enzyme
BAL: Broncoalveolar Lavage
CHGE: Covid Human Genetic Effort
COVAX: COVID-19 Vaccines Global Access
COVID-19: Coronavirus Disease 2019
COVID-19 H.G.I: COVID-19 Host Genetics Initiative
CSSE: Center for Systems Science and Engineering
DPP9: Dipeptidyl Peptidase 9
GWAS: Genome-Wide Association Study
ICU: Intensive Care Unit
IFNAR: Interferon-a/b receptor
IFN: Interferon
HLA: Human Leucocyte Antigen
HREs: Hexanucleotide Repeat Expansions
LoF: Loss-of-Function
OR: Odd Ratio
ORF: Open Reading Frame
SARS-CoV-2: Severe Acute Respiratory Syndrome Coronavirus 2
SLE: Systemic Lupus Erythematous
TLR: Toll-like Receptor
TMPRSS2: Transmembrane Serine Protease 2
VOC: Variant Of Concern
